# Application of Serological Tools and Spatial Analysis to Investigate Malaria Transmission Dynamics in Highland Areas of Southwest Uganda

**DOI:** 10.4269/ajtmh.15-0653

**Published:** 2016-06-01

**Authors:** Caroline A. Lynch, Jackie Cook, Sarah Nanyunja, Jane Bruce, Amit Bhasin, Chris Drakeley, Cally Roper, Richard Pearce, John B. Rwakimari, Tarekegn A. Abeku, Patrick Corran, Jonathan Cox

**Affiliations:** Faculty of Epidemiology and Population Health, London School of Hygiene and Tropical Medicine, London, United Kingdom; Faculty of Infectious and Tropical Diseases, London School of Hygiene and Tropical Medicine, London, United Kingdom; Makerere University College of Health Science, School of Biomedical Sciences, Department of Pathology, Kampala, Uganda; Abt Associates, Kampala, Uganda; Malaria Consortium, London, United Kingdom

## Abstract

Serological markers, combined with spatial analysis, offer a comparatively more sensitive means by which to measure and detect foci of malaria transmission in highland areas than traditional malariometric indicators. *Plasmodium falciparum* parasite prevalence, seroprevalence, and seroconversion rate to *P. falciparum* merozoite surface protein-1_19_ (MSP-1_19_) were measured in a cross-sectional survey to determine differences in transmission between altitudinal strata. Clusters of *P*. *falciparum* parasite prevalence and high antibody responses to MSP-1_19_ were detected and compared. Results show that *P*. *falciparum* prevalence and seroprevalence generally decreased with increasing altitude. However, transmission was heterogeneous with hotspots of prevalence and/or seroprevalence detected in both highland and highland fringe altitudes, including a serological hotspot at 2,200 m. Results demonstrate that seroprevalence can be used as an additional tool to identify hotspots of malaria transmission that might be difficult to detect using traditional cross-sectional parasite surveys or through vector studies. Our study findings identify ways in which malaria prevention and control can be more effectively targeted in highland or low transmission areas via serological measures. These tools will become increasingly important for countries with an elimination agenda and/or where malaria transmission is becoming patchy and focal, but receptivity to malaria transmission remains high.

## Introduction

In the east African highland areas, malaria transmission intensity generally decreases with altitude, often becoming heterogeneous as altitude increases, to a point where malaria is no longer transmitted.^1^–^6^ The main drivers behind these changes are thought to be a decrease in temperature and humidity that results in decreased mosquito vector density as altitude increases. However, clusters or hotspots of relatively high malaria transmission have been detected in highland areas, often associated with proximity to vector breeding sites such as forests, natural swamps, highland floodplains, or farmlands and pastures.^7^–^14^ Over a highland landscape, the heterogeneity in distribution of malaria can thus reflect microclimates suitable for vector breeding, coupled with differences in household structures or genetic factors.^15^–^18^

There is no standard definition of a malaria hotspot. The World Health Organization has previously defined foci of malaria as localities with continuous or intermittent epidemiological factors necessary for transmission.^19^ Bousema and others defined a hotspot as a geographical part of a focus where malaria transmission exceeds the average level in surrounding areas.^15^

Hotspots are likely to persist in highland areas unless interventions are targeted toward them.^8^,^20^,^21^ This of particular importance because these sinks could act as temporal “seeds” that propagate malaria outbreaks and epidemics should suitable conditions arise. Thus, identifying the precise location of hotspots toward which interventions can be targeted could potentially prevent epidemic outbreaks in addition to targeting individuals or areas that contribute disproportionally to malaria transmission.^22^

However, identifying hotspots of malaria in highlands is challenging. Standard measures such as entomological inoculation rates (EIRs) or parasite prevalence are more difficult to collect in low transmission areas due to very low numbers of either mosquitoes or infected individuals.^23^ In addition, both measures are affected by seasonality, so hotspots of transmission could be missed. Finally, the impact of increased malaria control interventions as well as the effects of interannual climate variability make understanding trends in malaria transmission in highland areas particularly difficult.

Conversely, antibody responses to some malaria parasite antigens have the potential to provide information about malaria transmission intensity over short or long periods of time. Drakeley and others estimated that merozoite surface protein-1_19_ (MSP-1_19_) antibodies persist for 49.8 years, reflecting cumulative exposure to malaria infection.^24^ By examining seroprevalence in different age groups and for the population as a whole, transmission intensity can be estimated for more recent as well as longer-term periods. Serological markers of transmission show greater sensitivity in low transmission areas, and as a measure are less affected by seasonality due to the longer duration of specific antibody responses.^24^

Our study used serological measures to assess malaria transmission at different altitudes in southwest Uganda. In addition, spatial analysis was used to determine whether hotspots of parasite-positive individuals are geographically similar to clusters of high antibody responses to MSP-1_19_.

## Materials and Methods

### Study area and population.

**Figure 1.**
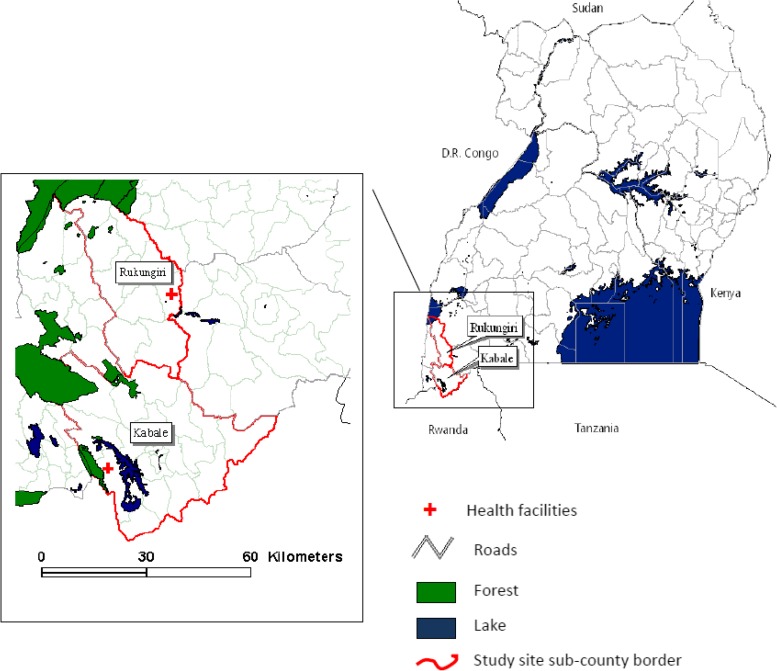
Study site subcounties and health facilities in Kabale and Rukungiri districts, southwest Uganda.

The ten villages included in the study were situated in the catchment areas of Kebisoni and Bufundi health facilities that are located in the highland and highland fringe districts of Kabale and Rukungiri in southwest Uganda. Bufundi health center is in a highland area at an altitude of 2,200 m and serves a population of approximately 18,000 that resides over altitudes of 1,700–2,200 m (Figure 1

). Kebisoni health center is in a highland fringe area at an altitude of 1,600 m and serves a population of 32,000 inhabitants residing over altitudes of 1,400–1,600 m.

Malaria is seasonal in both sites, with peaks in transmission in December (short rains) and April (long rains).

### Study design.

In August 2007 (dry season), a cross-sectional survey was implemented in ten villages over five altitudinal strata in southwest Uganda. Altitude was used as a proxy for transmission intensity, reflecting the relationship between altitude and lower temperatures that affects the intrinsic incubation period of the parasite in the mosquito. Two of the study strata were in the highland district of Kabale and three were in the highland fringe district of Rukungiri. The aim was to determine the prevalence of *Plasmodium falciparum* infections and to examine serological responses to *P*. *falciparum* MSP-1_19_, in a population residing along a transect of low to relatively high malaria endemicity.

Villages in health facility catchment areas were classified into five strata of malaria endemicity based on malaria incidence recorded by the health facility and using the health facility catchment population estimates. Local health teams verified the village categorizations based on their knowledge of malaria transmission intensity and village altitudes. One to three villages were included in each stratum depending on village size. The primary sampling unit for the study was the household. All households in the selected village that had members present and who consented to the study were sampled.

All consenting household members were tested for malaria parasite infection using the Paracheck Pf rapid diagnostic test (RDT) produced by Orchid Biomedical Systems (Goa, India). In addition, filter paper bloodspots were collected from all *P*. *falciparum* parasite–negative individuals, and were stored and prepared as described previously.^25^

#### Laboratory methods.

Antibodies to *P*. *falciparum* MSP-1_19_ were detected in the blood eluted from the filter paper blood spots by an indirect enzyme-linked immunosorbent assay (ELISA) using recombinant *P*. *falciparum* MSP-1_19_.^26^ Sera were tested at a single dilution (1:1,000) and a positive control curve of hyperimmune sera on each plate was used to standardize results between ELISA plates. Previous studies in the area showed that parasite-positive patients were significantly more likely to have traveled outside the area before presenting at health facilities, and that those who traveled were likely to be young men.^27^ We thus excluded *P*. *falciparum*–positive people from ELISA analysis during this study with the rationale that if those who were parasite positive were more likely to have traveled and of a certain age, that their inclusion into the sample would skew the age prevalence data. Restriction to parasite negatives gave a more normal age distribution for establishing the intensity of any ongoing background transmission.

### Statistical analysis.

Household and laboratory data were entered using Epi Info 2000 (CDC, Atlanta, GA); internal consistency checks and analyses were performed using STATA software (version 9; StataCorp, College Station, TX). Raw optical densities (ODs) were converted to percentages of a single point on the positive control curve. The cutoff between negative and positive was determined using maximum likelihood to estimate the parameters for a mixture model as described elsewhere.^26^,^28^ Age-specific seroprevalence data from parasite-negative individuals was fitted by age using maximum likelihood methods to obtain a seroconversion rate (SCR), which gives a serological measure of force of infection in the area, as described elsewhere.^24^,^25^ Rho (ρ), the seroreversion rate, was fixed based on previous estimates for MSP-1_19_ from similar epidemiological settings in Tanzania.^25^

### Spatial analysis.

To detect spatial clusters, all households included in the study were georeferenced and the Kulldorff spatial scan statistic was used to test whether seroreactivity to MSP-1_19_ and/or *P. falciparum* infections were distributed randomly over space, and if not, to identify significant spatial clusters.^29^,^30^ SatScan^©^ TM software (http://SatScan.org/) was used (Boston, MA). The spatial scan statistic uses a scanning window that moves across space. For each location and size of the window, the number of observed and expected cases is counted, and the window with the greatest ratio of observed to expected cases is noted. The numbers of expected cases are calculated by considering an even distribution of cases across the population. The statistical significance of the hotspot is evaluated by taking into account multiple tests for the many potential cluster locations and sizes evaluated.^31^ The scan statistic was calculated for two types of hotspots: 1) hotspots of *P. falciparum* parasites and 2) hotspots of MSP-1_19_ seroprevalence or immune responses.

For *P. falciparum* parasite hotspots, cases were people with *P. falciparum* parasites and controls were people who were *P. falciparum* parasite negative. For seroprevalence hotspots, log-transformed age-adjusted OD values were calculated. Adjustment for age was done by first log transforming antibody responses and then fitting Loess lines to determine at which point the relationship between age and log (Ab response) became non-linear. A linear regression model was run for each age group (0–11, 12–17, 17–22, 23–29, 30–52, and > 52 years) and residuals were used as age-adjusted antibody response.^32^ Residuals greater than zero were set as cases and those less than zero as controls for the scans.

Secondary clusters were set to not overlap the most significant cluster. For each location and size of the scanning window, a likelihood ratio test was conducted to test the hypothesis that there was an elevated rate of disease (or antibody response) when compared with the distribution outside. The window size and location with the maximum likelihood was defined as the “most likely” cluster (i.e., least likely to have occurred by chance). The distribution and *P*-value of the most likely and secondary clusters were determined by conducting Monte Carlo replications of the data set. To scan for small to large clusters, the maximum cluster size was set to 50% of the total population at risk. All data were examined at district level, that is, strata were split into the two highest altitudes (Kabale) and three lowest altitudes (Rukungiri). In the two highest strata, spatial analysis could only be undertaken with the serological data because of the very low number of *P. falciparum* parasite positive (*N* = 3) individuals detected. Maps were made using ArcGIS version 9 (Environmental Systems Research Institute, Redlands, CA).

### Ethical considerations.

Ethical clearance was obtained from the Ugandan National Technology and Science Council (HS-35) and the London School of Hygiene and Tropical Medicine (no. 3053). In addition, the blood sampling was demonstrated to the local chairmen and the executive committee of each village surveyed, and approval for the survey was sought. Informed consent was obtained from the head of the household for all human adult participants as well as any children under 18. Parasite-positive individuals were referred to the local health facility to receive free treatment.

## Results

A total of 2,125 individuals were sampled, all of whom were tested for parasite infections using Paracheck Pf RDT and 1,919 were tested for MSP-1_19_ using ELISA. Overall, the sample comprised 45.9% men and 54.1% women aged between 0 and 99 years (Table 1).

### Prevalence of *P. falciparum* malaria infection and MSP-1_19_ seroprevalence.

**Figure 2.**
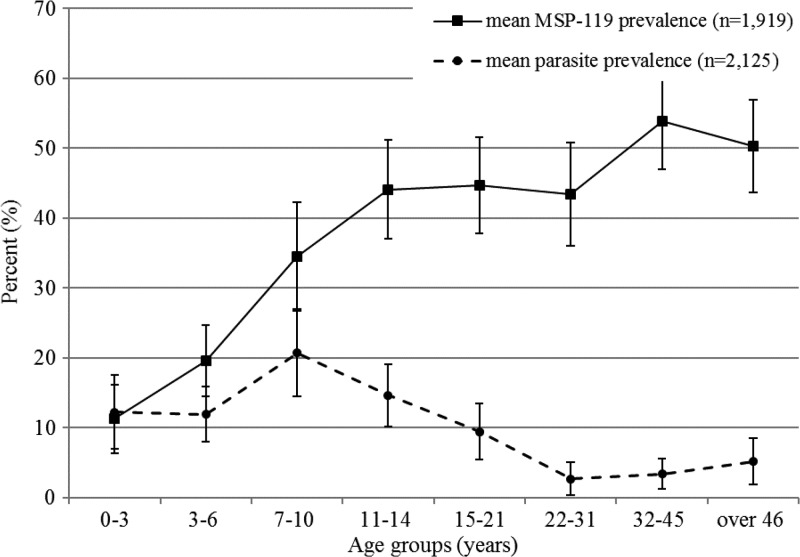
Prevalence of *Plasmodium falciparum* malaria infection and merozoite surface protein-1_19_ (MSP-1_19_) seroprevalence by age group.

**Figure 3.**
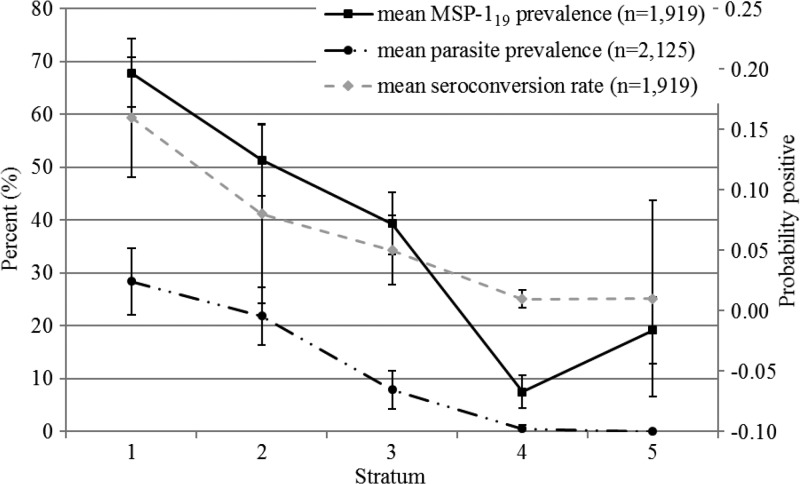
Prevalence of *Plasmodium falciparum* parasite prevalence and merozoite surface protein-1_19_ (MSP-1_19_) seroprevalence by strata.

Malaria infections were detected in 12.1% people (95% confidence interval [CI]: 10.0–14.2). Seroprevalence to MSP-1_19_ was 35.0% (95% CI: 32.1–7.9). The force of infection, calculated from MSP-1_19_ age–seroprevalence data, was estimated as a SCR of 0.04 per year across the study strata, correlating to an EIR of ∼1.3 (Figure 2

). Parasite and serological measures were highly correlated with altitude. *Plasmodium falciparum* prevalence decreased significantly as altitude increased (odds ratio [OR]: 0.5; 95% CI: 0.5–0.6) (Figure 2). Seroprevalence to MSP-1_19_ was significantly lower in subjects living in the highest altitude stratum (OR: 0.11; 95% CI: 0.07–0.19) compared with the lowest altitude (Figure 3). However, seroprevalence in stratum 5 was more than double that of stratum 4 indicating that factors other than altitude were influencing transmission there.

There was no significant difference between MSP-1_19_ seroprevalence and *P. falciparum* prevalence amongst men and women. Prevalence of *P. falciparum* infection was highest in 7- to 10-year olds. As would be expected, seroprevalence to MSP-1_19_ increased with age (Figure 2).

SCRs decreased from 0.16 (95% CI: 0.11–0.21) in stratum 1 (lowest altitude) to SCR 0.01 (95% CI: 0.00–0.01) in strata 4 and 5 see Table 2 and Supplemental Figure 1. The SCRs corresponded to EIRs of 31.7, 7.8, 3.3, 0.01, and 0.01 from the lowest to highest strata, respectively.

### Spatial distribution of *P. falciparum* parasite and MSP-1_19_ seroprevalence infections.

Age-adjusted *P. falciparum* seroreactivity was significantly increased in six clusters of households across the study sites: three in the upper two strata (Kabale) and three in the lower three altitudinal strata (Rukungiri).

#### Lower altitudinal strata (Rukungiri).

**Figure 4.**
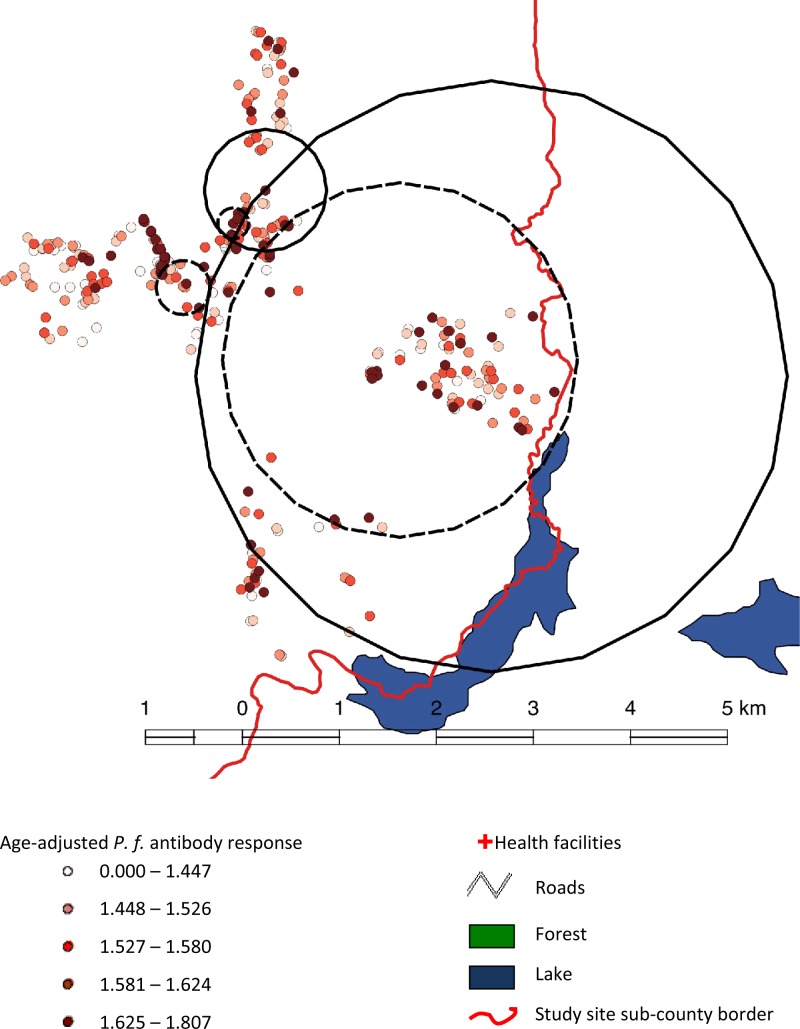
MSP-1_19_ seroprevalence (dashed) and *P.f.* infection (black line) ‘hotspots’ in highland fringe areas (Rukungiri).

Two clusters of *P. falciparum* infection were detected in the highland fringe strata of Rukungiri. One primary cluster of *P. falciparum* infection was detected with a radius of 3.1 km consisting of 155 households (*P* = 0.001) (Figure 4

). Further scans carried out at village level detected one cluster with a much smaller radius of 0.60 km in which there were 40 households (*P* = 0.04).

A primary serological cluster was detected in Rukungiri, which consisted of 11 households (Figure 4) with a radius of 0.16 km at an altitude of 1,470–1,539 m (*P* = 0.001). A pond lies in the middle of the primary cluster, which is also flanked (outside the cluster) by two protected springs (0.35 km and 0.46 km from the cluster center) and another pond (0.35 km).

Two secondary serological clusters were also detected across the three lower altitudinal strata, the largest of which were close to the lake and consisted of 93 households and spanned a radius of 1.83 km (Figure 4) (*P* = 0.004). The other secondary cluster detected consisted of 16 households and spanned 0.28 km (*P* = 0.006). The center of the cluster was 0.35 km away from a forested area and within the cluster there were three unprotected wells and a stream.

Parasite infection and seroprevalence clusters overlapped to a large extent in the fringe highland strata. The largest serological cluster lay completely within the primary parasite cluster, but had a far smaller radius and number of households. One of the secondary serological clusters fell with the secondary parasite infection cluster, but was again far smaller in radius. One additional secondary serological cluster fell completely outside either of the parasite infection clusters.

#### Upper altitudinal strata (Kabale).

As only three parasite-positive subjects were found in the two highland strata in Kabale District, spatial analysis was not possible. The three cases were in two different villages: two in a village closest to Lake Bunyonyi and one in a village 2 km to the north at altitudes of 2,096 and 2,153 m.

**Figure 5.**
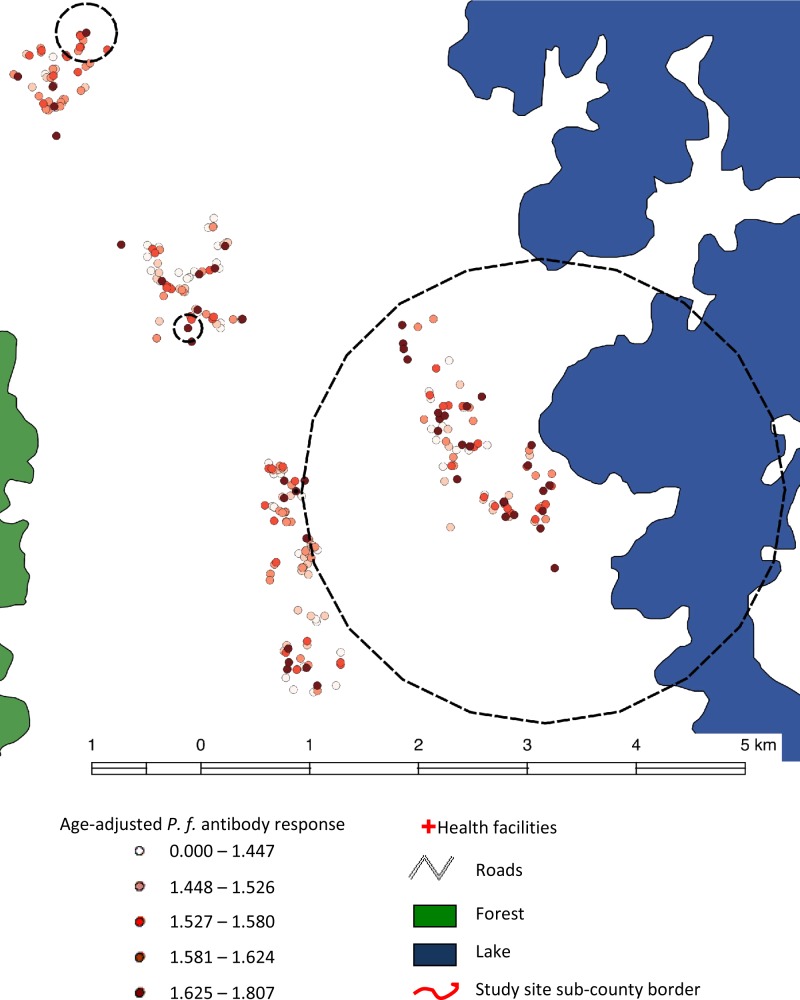
MSP-1_19_ seroprevalence ‘hotspots’ in highland areas, Kabale.

The primary cluster in the upper two strata included 95 households and had a radius of 2.2 km (*P* = 0.001) (Figure 5

). The cluster included the village closest to Lake Bunyonyi which is within 2 km of the lake and situated between altitudes of 2,107 and 2,180 m. Two secondary clusters were also detected, consisting of 13 (*P* = 0.001) and four (*P* = 0.001) households in two different villages. None of these villages were situated close to mapped open sources of water such as lakes or ponds.

## Discussion

A cross-sectional survey was undertaken across five strata of different malaria endemicity in the highland areas of southwest Uganda. Both parasite prevalence and serological responses to *P*. *falciparum* MSP-1_19_ were used to investigate transmission dynamics. Results indicate that parasite prevalence decreased with increasing altitude, with only three parasitemic subjects found in the two highest altitude strata. SCR, which has been shown to correlate with EIR,^24^,^33^ decreased with altitude except at the very highest altitude where it was greater than that for the next lowest stratum. Seroprevalence in this highest stratum was driven in part by high seroprevalence among 7- to 15-year olds in one of the villages closest to the health facility. One possibility is that the decline in drug efficacy that began around 15 years before the survey resulted in increased seroprevalence responses in this age group, whereas with adults this would not have been the case. Less efficacious drugs could result in lack of clearance of parasites and therefore higher exposure of the immune system to parasites resulting in a stronger immune response. The sudden drop in SCRs 7 years ago could also be explained by the introduction of a combination therapy chloroquine and sulfadoxine-pyrimethamine, followed by the introduction of ACTs. Alternatively, a higher seroprevalence in this age group could be related to internal migration to lower altitudes (and thus higher transmission intensity) for primary or secondary education. Historically, children in Uganda were sent to boarding schools for education (Gould, 1975) and this continues to an extent today (C. A. Lynch, unpublished data). Overall, results suggest that while altitude is a good proxy for transmission intensity, other factors such as migration may influence transmission in the area.

Significant hotspots were detected for both *P. falciparum* infections and seroreactivity to MSP-1_19_ in the highland and highland fringe sites. In the highland fringe sites, clusters of parasite prevalence and MSP-1_19_ overlapped, but a greater number of clusters of smaller diameter were detected using age-adjusted seroreactivity. A hotspot of seroreactivity was detected where no parasite prevalence was measured, and at a surprisingly high altitude of ∼2,200 m suggesting that transmission is ongoing in that area, despite no current infection being detected. This may have been because the survey was undertaken during the dry season.

It is not possible to determine whether the hotspots detected in this study are stable or not. Few studies have examined the stability of hotspots over time. Those that have used multiple years of either malaria infection or clinical episodes to identify clusters that predicted future hotspots.^8^,^15^,^34^ Mosha and others found that clusters with high MSP-1_19_ seroprevalence were at lower risk of infection suggesting some protection at the neighborhood level.^35^ The same study found that malaria infection clusters were predictive of future hotspots and that hotspots of seroprevalence (in this case apical merozoite antigen-1) were predictive of future malaria infection. Mosha's findings indicate that hotspots of seroprevalence are relatively stable for several years.^35^ Applied to our findings, this would mean that the hotspots we identified could possibly be used to more effectively target interventions in the coming 2–3 years.

Our results confirm findings from studies that demonstrate altitude to be highly correlated with malaria infection in Uganda and that serological responses to MSP-1_19_ can be used as a proxy for longer-term malaria transmission, which allows for historical hotspots to be identified.^4^,^5^,^24^,^36^–^41^ Previous studies in southwest Uganda have shown that transmission intensity in the highland areas is generally low but increases sharply below 1,500 m with the exception of hotspot areas around lakes or other types of breeding sites.^4^,^36^,^38^,^42^–^45^ Nevertheless, *P. falciparum* infection measured in this study was still relatively high in lower altitudinal strata of Rukungiri compared with previous studies, even though the survey was undertaken in the dry season. For example, parasite prevalence in 2- to 9-year olds from this study is far higher for those reported from Jelliffee's study in 1957 (prevalence rate [PR]: 1.6%) or Langi and Lalobo's study in the same district in 1992 (PR: 2.7%).^42^,^46^ However, results are comparable to those found by Killian and others in Kabarole in 1994, just north of Rukungiri (PR 27.8% at 1,530 m), although those results were an average of both wet and dry season rates.^4^ Differences could be attributed to spatial variation in transmission. Alternatively, they could be as a result of changing epidemiology in the southwestern highlands in general. Previous studies took place sometimes up to five decades before our research. Since that time, the highlands have undergone deforestation and farming-related changes in land use both of which can affect malaria transmission patterns.^47^ Changes in climate in east Africa are also debated as to whether they have had a negative impact on malaria transmission in the highlands specifically.^48^–^51^

In addition, previous studies used microscopy to detect malaria parasites whereas our study used RDTs. Abeku and others have demonstrated that RDT specificity varies considerably by month of test, age of patient, and presence or absence of fever during consultation in this area of Uganda.^52^ Their findings indicate that RDTs had a high false-positive error rate (up to 30%) in the areas of our study, which may mean that the relatively high prevalence detected in Rukungiri could have been as a result of false positives rather than true parasite infections.

Three of the six clusters detected (including the largest clusters) were near open water bodies such as lakes or ponds that confirms findings from other studies in highland settings that showed that clusters of *P. falciparum* parasite prevalence existed along lakeshore areas.^53^ Entomological findings in the same area found that both *Anopheles* *gambiae* s.s., the dominant vector in the area, and *Anopheles funestus* preferred open water bodies with the latter species playing an important role in transmission around the highland lakes.^6^

There are several limitations to the study. First, we stratified villages in the study sites by using incidence rates from health facilities in the area. However, it is possible that villages further away from health facilities were classified as lower endemicity when the lower number of malaria cases was as a result of their distance from the facility rather than true endemicity. This would have resulted in a misclassification of village endemicity relative to others along the altitudinal transect. Our survey undersampled men that could have led to an underestimation of overall *P. falciparum* prevalence and seroprevalence, if they were absent because of travel outside the area to places of higher malaria transmission intensity.^27^ We also excluded parasite-positive individuals, which could have led to an underestimation of SCRs overall. Finally, as previously mentioned, using RDTs in this area could have led to a high false positivity rate and an overestimation of parasite prevalence.

Regardless of these limitations, the study findings have multiple implications. First, results confirm the existence of hotspots of malaria transmission at extremely high altitudes in Uganda. Although these were documented previously in the 1940s at slightly lower altitudes in the same area,^36^,^54^ their continued existence in the presence of increased control measures suggests that other factors, such as migration, are potentially driving increased seroprevalence rates. Lynch and others have previously described the association between malaria incidence and travel in the highest altitude strata this should be explored further in terms of seroepidemiology and migration.^27^ Our findings also further confirm the use of seroprevalence as a useful diagnostic to assess exposure to infection, particularly in higher altitude areas (lower transmission intensities) where parasite prevalence is often not detectable with conventional diagnostics.

In an era of malaria elimination and in the context of significantly reduced financial resources, methods to accurately identify and target areas at risk of malaria transmission are crucial. As malaria burden decreases, malaria infections are likely to become more spatially heterogeneous because of differences in acquired immunity related to the clustering of malaria transmission in highland areas, and seasonal expansion of hotspots.^15^ In addition, internal circular migration from, and back to, highland areas and emergence of drug resistance, often associated with highlands, adds more complexity to the epidemiological pattern of malaria infections in these areas.^27^,^55^–^58^ Different types of clusters, stable asymptomatic infections, and unstable febrile cases, were detected by Bejon and others in Kenya demonstrating that serological tools could not only identify hotspots but could also be used to disaggregate types of hotspots clusters of malaria transmission risk in the longer term.^34^ Although the malaria burden reduces with control, the risk of malaria transmission remains and thus, the risk of resurgence.^59^ Countries aiming for malaria elimination require a detailed understanding of the current and potential intensity of malaria transmission should control measures be reduced. Using seroprevalence as a measure of transmission dynamics presents a powerful tool to help achieve longer-term malaria elimination goals.

## Supplementary Material

Supplemental Figure.

## Figures and Tables

**Table 1:** Summary table of population characteristics including age, gender, socioeconomic status, and residency

	*n*	Stratum 1 (lowest altitude)	Stratum 2	Stratum 3	Stratum 4	Stratum 5 (highest altitude)
Age group (years)	%					
< 1	52	2.9	3.2	3.7	0.8	2.0
1–4	301	16.0	12.1	15.6	14.1	14.1
5–14	748	38.2	30.8	34.6	38.5	34.7
15−44	756	31.4	39.9	36.4	33.9	35.6
> 45	266	11.5	14.0	9.8	12.7	13.6
Gender						
Male	898	41.6	43.3	39.5	40.5	46.0
Female	1,225	58.4	56.7	60.6	59.5	54.0
SES						
SES 1 (poorest)	530	29.5	23.8	12.8	37.6	16.8
SES 2	532	32.6	27.8	17.7	24.7	20.5
SES 3	531	193	28.4	32.1	17.2	29.5
SES 4 (least poor)	532	18.5	20	37.3	20.5	33.2
Residency						
Born in subcounty	1,739	91.4	87.0	81.0	76.6	73.0
Born outside subcounty	386	8.6	13.0	19.0	23.4	27.0

SES = socioeconomic status.

**Table 2:** Risk of being MSP-1_19_ or *Plasmodium falciparum* positive compared with highest altitude stratum (5) and estimated EIR

Study strata	Risk of seroprevalence (MSP-1_19_) (OR)	Risk of parasite prevalence (OR)	SCR (*λ*)	Estimated EIR
Stratum 1	8.9 (5.4–14.7)	76.7 (20.5–286.4)	0.15 (0.11–0.21)	31.7
Stratum 2	4.4 (2.7–7.2)	54.0 (14.5–202.2)	0.08 (0.06–0.11)	7.8
Stratum 3	2.7 (1.7–4.4)	16.5 (4.2–65.6)	0.05 (0.03–0.08)	3.3
Stratum 4	0.3 (0.2–0.6)	0	0.01 (0.00–0.01)	0.1
Stratum 5	1.00	1.00	0.01	0.1

EIR = entomological inoculation rate; MSP-1_19_ = merozoite surface protein-1_19_; OR = odds ratio; SCR = seroconversion rate.
