# Supplementary material for: Application of Serological Tools and Spatial Analysis to Investigate Malaria Transmission Dynamics in Highland Areas of Southwest Uganda
Source: Am J Trop Med Hyg. 2016 Jun 1;94(6):1251–8. doi: 10.4269/ajtmh.15-0653 (PMC4889741; doi:10.4269/ajtmh.15-0653)
Supplement: Supplementary file 1 [file SD2.pdf]

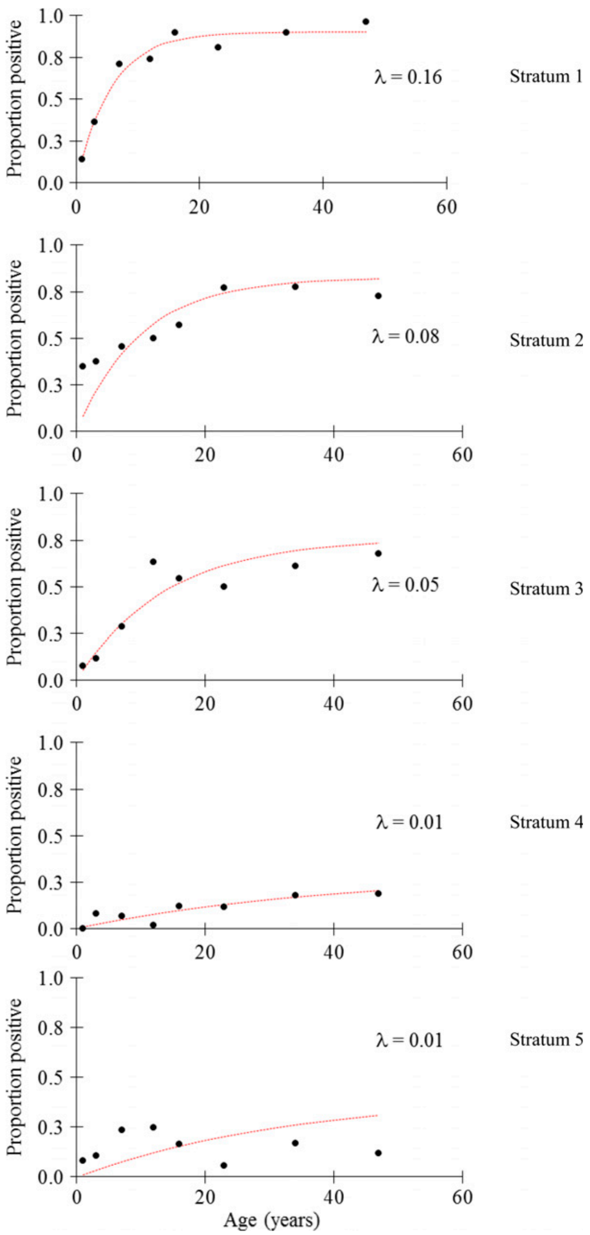

SUPPLEMENTAL FIGURE 1. Seroprevalance curves for *Plasmodium falciparum* merozoite surface protein-1_{19} (MSP-1_{19}) antigens for each stratum, Uganda.
